# Growing body of evidence supports intrauterine insemination as first line treatment and rejects unfounded concerns about its efficacy, risks and cost effectiveness

**DOI:** 10.5935/1518-0557.20180073

**Published:** 2019

**Authors:** Gulam Bahadur, Roy Homburg

**Affiliations:** 1 Reproductive Medicine Unit, North Middlesex University Hospital, Old Admin Block, Sterling Way, London N18 1QX, UK; 2 Homerton Fertility Unit, Homerton University Hospital, Homerton Row, London E9 6SR,UK

**Keywords:** IUI, IVF/ICSI outcome, assisted reproduction, cost-effectiveness, ethics

## Abstract

IUI has been practiced for five decades but only three unconvincing trials attempted to demonstrate the superiority of IUI over sexual intercourse (SI). In the absence of evidence of its effectiveness, the National Institute for Clinical Excellence (NICE) recommended IVF over IUI after 2 years of unprotected SI. High-quality recent data in well-constructed studies suggest that biases against IUI procedures and in favour of IVF are invalid. It is unethical to continue to misinform patients and stakeholders. The well-constructed randomised controlled trials (RCT) show IUI procedure to be efficient, with minimal risk, and above all improved cost-effectiveness when compared to IVF for live birth. IUI as first-line treatment should be offered to most patients, while funding agencies and stakeholders need to be urgently informed of the cost-benefit in offering IUI. Fertility clinics, IVF interest groups, and regulatory bodies should amend their patient information and guidance to state that IUI should be the first line treatment and that IVF should be offered only when essential. Reappraising and promoting IUI based on evidence enhances patient autonomy, choices, and trust, while allowing the fertility industry to operate within an ethical and acceptable framework not seen as exploitative toward vulnerable patients.

## BACKGROUND

IUI as first line treatment option is highly desirable for couples, easily accessible and practised worldwide. The procedure is also well accepted on account of its simplicity (Bahadur *et al*., 2016a). Questions over the efficacy, risks and cost effectiveness of IUI are occasionally raised in reviews and guidelines based on the analysis of evidence, as in the case of the controversial and much disputed National Institute for Clinical Excellence (NICE) 2013 guidelines on IUI. Overuse of expensive IVF procedures becomes a contentious issue with regards to public funding when most public bodies around the world face budgetary limitations (Bahadur *et al*., 2016b).

For the 12^th^ consecutive year, European data on IUI using husband/partner semen (175,467 cycles) from 27 countries including France, Italy, Spain, Belgium and Poland, revealed that in 24 countries the mean live birth rate (LBR) per cycle was 8.6%, while twin and triplet birth rates associated with IUI were 9.5% and 0.6%, respectively. When all IVF cycles were considered, the clinical pregnancy rates (PR) per aspiration and per transfer were 29.6% and 34.5%, with a total multiple LBR of 18.0%. The proportions of singleton, twin and triplet deliveries after IVF and ICSI (added together) were 82%, 17.5% and 0.5%, respectively (European IVF-monitoring Consortium, 2017). In contrast to the extensive cross analyses on outcomes for IVF/ICSI cycles, the report places little emphasis on IUI cycles (European IVF-monitoring Consortium, 2017) despite its benefits and against the ill-constructed NICE guidelines excluding IUI as a treatment modality (Bahadur *et al*., 2017), which only considered IUI/CC (25mg). The method of reporting varied between countries. Some contained severe limitations and should therefore be interpreted with caution. The data were incomplete and collected using different methods. A number of countries were unable to provide relevant data such as number of initiated cycles and deliveries.

The Cochrane review on IUI for unexplained subfertility (Veltman-Verhulst *et al*., 2016) identified three relevant RCTs (Deaton *et al*., 1990; Steures *et al*., 2006; Bhattacharya *et al*., 2008) involving a total of 690 couples (mean female age of 33 years) trying to conceive for an average of 2 to 4 years. One study compared IUI to timed SI with cycle monitoring (Deaton *et al*., 1990), while the other two compared IUI with SI without any medical co-interventions (Steures *et al*., 2006; Bhattacharya *et al*., 2008). The odds ratio (OR) of clinical pregnancy for IUI without COS compared to SI was 1.53 (95% CI: 0.88-2.64), while the pooled OR of clinical pregnancy for IUI with COS compared to SI was 1.00 (95% CI: 0.59-1.67). The OR for multiple pregnancy was 0.50 (95% CI: 0.04-5.53) for IUI without COS compared to SI and 2.00 (95% CI: 0.18-22.34) for IUI with COS compared to SI. One RCT with 102 women offered same-dose FSH stimulation and who developed two or three follicles were randomised 3:1 to IUI (n=33) or IVF (n=10) (Elzeiny *et al*., 2014). IUI or IVF was performed 36h after hCG administration with single or double embryo transfer on day two yielded clinical pregnancy rates (40% vs. 12%, *p*=0.04) and live birth rates (40% vs. 6%, *p*=0.01) favouring IVF over IUI. The data appears to suggest there is a disproportionate loss of pregnancies in the IUI group compared to the IVF group. While IUI outcomes resided towards the low end of what most practitioners experience, it is difficult to comprehend how they could justify dismissing IUI.

### Past status of IUI

Previous ESHRE workshop reports presented generalised, albeit bleak IUI guidance, stating that IUI treatment requires ovarian stimulation to achieve modest results, but the high multiple pregnancy rates meant that it is no more than a poor substitute for IVF treatment. Further statements included;” *IUI in stimulated cycles was effective only in patients with more than 3 years duration of infertility but is associated with a significant rate of higher-order multiple births; although IUI treatment is cheaper and less demanding on the patient, IVF is the most effective treatment for infertility; IUI/ovarian stimulation: modest effect and risks of multiple pregnancy and OHSS whereas IVF offered a 7-fold higher likelihood of pregnancy*” (ESHRE Capri Workshop Group, 2009). These recommendations, however, were made in the absence of proper management trials and almost absent IUI live birth data. This raises profound questions on the bias allied to IVF practices, and whether fair and ethical first line treatment options are offered to patients (Bahadur *et al*., 2016a).

A later Cochrane and Capri review (Pandian *et al*. 2012; ESHRE Capri Workshop Group, 2015) identified three trials comparing ovarian stimulation plus IUI with IVF. Due to different protocols with significant heterogeneity, the data from only two of these (small) studies, comparing three cycles of ovarian stimulation plus IUI with one cycle of IVF, could be aggregated. The pooled odds ratio for IUI was 1.09 (95% CI 0.74-1.59), suggesting no clear benefit associated with either treatment.

### The current evidence for IUI

Recent evidence dispels the prevailing prejudices surrounding IUI treatment. Two randomised controlled trials reported at ESHRE 2017 (Brown, 2017) further prompt a change in the NICE guidelines (although these are only applicable to UK NHS centres). The first RCT with 201 couples with 3-4 years unexplained infertility randomised towards three cycles of IUI (CC) or expectant management (Farquhar *et al*., 2017; 2018) revealed a three-fold improvement in outcome in live birth rate from 31% and 9%. Another RCT supported IUI as first line treatment using CC over low dose FSH (Danhof *et al*., 2017a;b). The second RCT involved 24 centres in the Netherlands (Brown, 2017), 369 women having IUI stimulated with FSH and 369 women with CC; 31% (n=113) were pregnant with IUI-FSH; 26% (n=97) became pregnant with IUI-CC; 3 women (1%) had multiple pregnancy following IUI-FSH; 8 (2%) women had multiple pregnancy following IUI-CC (NS). They also reported 48 of 210 pregnancies (23%) by natural conception (in line with the natural conception rate) suggesting ‘expectant management’ might not be such a hopeless alternative. The findings are similar to an earlier RCT (Nandi *et al*., 2017). The overall conclusion is that ‘*IUI with CC may be offered to couples with unexplained infertility as a safe and effective treatment*.’ The NICE recommendations were based on just two trials in unexplained infertility, one of which included IUI without stimulation (Brown, 2017). Similar conclusions were drawn about retracting the NICE guidelines on IUI (Brown, 2017; Bahadur *et al*., 2017). The PRISM consortium also supported IUI over expectant management in unexplained subfertility where the fecundity ratio for conceiving after IUI compared to expectant management was 1.56 (95% CI: 1.20-2.03). IUI provides higher pregnancy rates compared to expectant management and thus must remain as a first-line treatment for couples with unexplained subfertility.

Few studies report success rates in fertility treatments across a couple’s complete fertility treatment history, across clinics, evaluating live births after insemination, ART and natural conceptions. A study looked into the Danish ART Registry and Medical Birth Registry for live births (2007-2010). Sub-fertile couples were followed for 2 years (N=19 884), 3 years (N=14 445) and 5 years (N=5165), or until their first live birth. Cumulative live birth rates were estimated 2, 3 and 5 years from the first treatment cycle, in all women, including dropouts. In women starting treatment with IUI (N=3028), 35% delivered after IUI within 5 years, 24% delivered after a shift to IVF/ICSI treatments and 17% delivered after natural conception. Overall, more than 50% of the women starting IUI did not need demanding IVF. Within 5 years from starting treatments with ART (N=2137), 53% delivered after ART, 11% delivered after natural conception, and 0.6% delivered after IUI (Malchau *et al*., 2017). While most of the deliveries occurred within 2 years, the data also confirmed that 52% of women entering fertility treatment did not require IVF procedures (Malchau *et al*., 2017). The lack of IUI pregnancy rate optimisation has been of concern especially when non-evidence based expensive add-on techniques are applied for IVF. Curiously, utilising a double IUI within a cycle had been dismissed (Cantineau *et al*., 2003). However, this Cochrane review actually stated that there was a significant benefit to using double IUI within a cycle (OR: 2.0; 95% CI: 1.07-3.75; *p*<0.03). Likewise, significantly positive pregnancy outcomes were seen with endometrial scratching within the follicular phase of 1,871 IUI cycles (OR: 2.27; *p*<.00001).

The overuse of IVF procedures is increasingly seen as a major risk factor in publicly funded fertility treatments (Bahadur *et al*., 2016b). Although current data does not reveal a multiple birth problem with IUI, well-managed IUI with strict cancellation policies would obviate the risk for multiple births. A major practical innovation in IUI has been applied for overcoming severe male factor infertility by using consecutive ejaculation to boost the total motile sperm numbers, effectively cancelling out the oligozoospermic effect and thereby improving IUI pregnancy outcomes (Bahadur *et al*., 2016c). It means that males with severe male factor must be explored for their potential to gain more motile sperm through consecutive ejaculates before embarking on IVF procedures. Overcoming male factor infertility in IUI has also been observed when performing double inseminations within a cycle (Ghanem *et al*., 2011).

In a RCT, ovarian stimulation with low-dose hMG was superior to CC in IUI cycles with respect to clinical pregnancy rate. Ovarian stimulation with hMG yielded a higher clinical pregnancy rate (hMG 48/334 (14.4%) versus CC29/323 (9.0%), relative risk (RR) 1.6 (95% confidence interval (CI) 1.1-2.4)). The LBR was higher (hMG46/334 (13.8%) versus CC28/323 (8.7%), RR1.6 (95% CI 1.0-2.4), low and comparable multiple live birth rate (hMG 3/46 (6.5%) versus CC 1/28 (3.6%), *P*. 0.99) (Peeraer *et al*., 2015). They recommended that IUI combined with low-dose gonadotropins is the treatment of choice for patients with indication for IUI treatment. In another RCT, after treatment with gonadotropin, clomiphene or letrozole, clinical pregnancies occurred in 35.5%, 28.3%, and 22.4% of the cycles, and live births in 32.2%, 23.3%, and 18.7%, respectively; pregnancy rates with letrozole were significantly lower than the rates with standard therapy (gonadotropin or clomiphene) (*p*=0.003) or gonadotropin alone (*p*<0.001), but not with clomiphene alone (*p*=0.10) (Diamond *et al*., 2015). In a RCT with 602 couples on IVF-SET, IVF-modified natural cycle (194) and IUI-COH (207), the birth of a healthy child occurred in 104 (52%) couples in the IVF-SET group, 83 (43%) in the IVF-modified natural cycle group, and 97 (47%) in the IUI-COH. This corresponds to a risk, relative to IUI-COH, of 1.10 (95% confidence interval 0.91 to 1.34) for IVF-SET and 0.91 (0.73 to 1.14) for IVF-modified natural cycle. All groups had similar outcomes, with low multiple pregnancy rates (Bensdorp *et al*., 2015). The HFEA IUI data on pregnancy rate per cycle in the <35 age group in 2010 to 2015 improved from 9.12% to 15.17%. This contrasts sharply against the 4-7%/cycle considered for the weakly evidenced based NICE guidelines (Bahadur *et al*., 2016a; 2017). The Centre for Evidence-Based Medicine in Oxford independently provided evidence that IUI was effective on LBR (odds ratio was 1.95 (1.10 to 3.44) (95% CI)) when compared with intercourse or expectant management in a stimulated cycle (Heneghan *et al*., 2016). This study performed independently of fertility practitioners also highlighted how statistically superior IUI is when placed in the context of promoting expensive add-on IVF techniques such as endometrial scratching or time lapse embryoscopy for which little evidence-based support exists (Heneghan *et al*., 2016). Better quality evidence to help patients make informed choices is necessary and it has been debated that desired results might be obtained through bias in study selection (Alikani *et al*., 2018).

The moral concerns against selectively promoting expensive IVF over IUI particularly highlights invalid reasoning and unproven effectiveness, while exposing patients to risks and economic burdens (Tjon-Kon-Fat *et al*., 2016).

### Financial consideration

In an early economic evaluation of IUI in which pregnancy rates were already substandard to UK (Bahadur *et al*., 2016a), outcomes showed costs for IUI of £98 per cycle, compared to £0 for SI (Wordsworth *et al*., 2011). The authors unsurprisingly concluded that IUI was an ineffective and costly treatment. This analysis was widely regarded as poor, since pregnancy outcomes were typically half of the UK average. However, current analyses characterised IUI as cost effective. IUI is less expensive than IVF, since the mean cost per live birth for IVF is 7187 Euros versus 5070 Euros for IUI. The cost effective incremental ratio per live birth for IVF-SET compared with IUI-COH was €43,375, reflecting the additional costs necessary to achieve one additional healthy child in IVF-SET versus IUI-COH (Tjon-Kon-Fat *et al*., 2015). Another strategy dictates that postponing IVF by 1 year might be a cost-effective measure forward, but this also depends on prognosis. The cost-effective CE ratio, i.e. the incremental costs of immediate versus delayed IVF per extra live birth, is the highest (range of €15 000 to >€60 000) for couples with unexplained infertility and for them it depends strongly on female age and duration of infertility, whilst being lowest for endometriosis (range 8000-23 000) and, for such patients, only slightly dependent on female age and duration of infertility (Eijkemans *et al*., 2017). While it is generally recognised that CC is a less expensive drug than gonadotropins, it is actually more cost effective to achieve pregnancy with gonadotropin (Peeraer *et al*., 2018).

Multiple birth is a well-known major risk of IVF procedures and hence a major drive for SET procedures. The cost of fetal reduction has never been factored in for IVF procedures. Importantly, the actual costs of multiple births and associated problems have so far been omitted in IVF costing as has its impact on national healthcare and human costs. Recent financial analysis shows a significantly higher total cost (ART treatment, pregnancy follow-up, delivery, child cost until the age of 2 years) for multiple births (both children: mean €43,397) than for singleton births (mean: €17,866) (*p*<0.0001). A 50% reduction in multiple LBR resulted in a significant 13% reduction in hospital care costs (Peeraer *et al*., 2017). Financial analysis of fertility treatments and outcomes is clearly a complex endeavour with several levels, on the impact cannot be assumed to be the same for IVF and IUI without having a measure of the size of the problem.

### Risk implication of multiple births within IUI and IVF

IUI has been liberally blamed for multiple births although such data is usually absent - in the UK, the HFEA does not collect live birth data for IUI. NICE assumed that IUI and IVF yield equal numbers of multiple births despite the absence of LBR data for IUI. The UK NICE costing (NICE, 2013) adopted a quality adjusted life years (QALYs) model to allow comparisons between infertile women and other clinical conditions. This approach is controversial because infertility care values cannot be easily captured in QALYs (Devlin & Parkin, 2003). The model fails to address the complexities faced by several stakeholders involved, as well as the size of the problem for society. The argument on multiple births lingers on a discourse suggesting eSET has eradicated multiple births in IVF procedures. Evidence from a multicentre study has shown that twin pregnancy rates can be very low in an IUI programme (much lower than in most IVF programmes worldwide) with a reasonable IUI pregnancy outcome (Bensdorp *et al*., 2015).

On average, 1 in 10 IVF pregnancies is a multiple pregnancy, compared to 1 in 80 for women who conceive naturally. With approximately 19,000 IVF babies born in the UK in 2014, this contributes significantly to the rate of multiple births (NOS, 2017). In January 2009, the HFEA introduced a policy to minimise the risk of multiple births from IVF treatment. This allowed centres to develop their own strategy - the aim was to reduce the UK IVF multiple pregnancy rate to 10% over a period of years.

The UK facts for multiple births where data is available is as follows; 1 in 80 births following natural conception in the UK are multiples; 1 in 4 births after IVF in the UK result in either twins or triplets (incl. ICSI); 40% of IVF babies are twins. The number of multiple babies has risen significantly: in 1995 just over 2600 IVF babies were born as part of a multiple birth; in 2003 more than 3700 IVF babies were born as part of a multiple birth - a rise of more than 41%; 126 IVF babies die each year as a consequence of having been born in a multiple birth. Of these 51 are stillbirths, 42 died within the first week of life, and 33 died later in the first year of life. These figures do not include miscarriages or fetal reduction procedures (Oakley & Doyle, 2006; NOS, 2017). There is increased risk (ranging 2-18 times higher) for babies and mothers associated with twin and triplet births for being born prematurely, death in the first week of life, cerebral palsy, while mothers face similarly increased risk of pre-eclampsia, diabetes, coronary heart disease, and death for cardiovascular causes (Sattar & Greer, 2002).

Although fertility clinics market themselves on league table type success rates, they are less clear about the issues derived from the multiple births produced in each clinic. More importantly, in the UK multiple births remain invisible to specific IVF clinics. Fertility clinics remain unaccountable to multiple birth contributions and do not have to make any financial contribution towards immediate care with obstetric complications. Multiple births in IVF have, therefore, been seriously overlooked in terms of the human and economic costs and one might question why financial analyses have never factored in the cost effectiveness of IVF or IUI procedures.

## CONCLUSION

All recent high-quality evidence suggests old conclusions should be updated. Prejudices surrounding IUI and in favour of IVF as being substandard or fraught with risks of multiple births is completely unfounded and needs to be reappraised on evidence. Costing analyses so far have failed to capture the serious health and human cost of higher order births stemming from IVF procedures. It raises questions on conflict of interests, but also demands that we develop a moral and ethical perspective when offering first line treatment options to patients in a balanced and fair manner. Only the financial interest related to offering expensive IVF procedures stand in the way of an exciting new era of growth for IUI procedures, which can be overcome by prudent government funding policies where funding exists. Greater use of low cost IUI treatment will also increase the chance to fund IVF treatment when needed. Public bodies and IVF interest groups have a duty of care to accurately inform patients, the public, funding bodies and stakeholders of the benefits, efficacy and cost-effectiveness of IUI before embarking on expensive IVF procedures.
